# Cooperation between HMGA1 and HIF-1 Contributes to Hypoxia-Induced *VEGF* and *Visfatin* Gene Expression in 3T3-L1 Adipocytes

**DOI:** 10.3389/fendo.2016.00073

**Published:** 2016-06-27

**Authors:** Sebastiano Messineo, Anna Elisa Laria, Biagio Arcidiacono, Eusebio Chiefari, Raúl M. Luque Huertas, Daniela P. Foti, Antonio Brunetti

**Affiliations:** ^1^Department of Health Sciences, University “Magna Græcia” of Catanzaro, Catanzaro, Italy; ^2^Department of Cell Biology, Physiology and Immunology, Instituto Maimónides de Investigación Biomédica de Córdoba (IMIBIC), Hospital Universitario Reina Sofía (HURS), CIBERobn and ceiA3, University of Córdoba, Córdoba, Spain

**Keywords:** hypoxia, HMGA1, HIF-1, obesity, Visfatin, VEGF, adipocytes, gene transcription regulation

## Abstract

The architectural transcription factor high-mobility group AT-hook 1 (HMGA1) is a chromatin regulator with implications in several biological processes, including tumorigenesis, inflammation, and metabolism. Previous studies have indicated a role for this factor in promoting the early stages of adipogenesis, while inhibiting adipocyte terminal differentiation, and decreasing fat mass. It has been demonstrated that hypoxia – through the hypoxia-inducible factor 1 (HIF-1) – plays a major role in triggering changes in the adipose tissue of the obese, leading to inhibition of adipocyte differentiation, adipose cell dysfunction, inflammation, insulin resistance, and type 2 diabetes. To examine the possible cooperation between HMGA1 and HIF-1, herein, we investigated the role of HMGA1 in the regulation of *Visfatin* and *VEGF*, two genes normally expressed in adipose cells, which are both responsive to hypoxia. We demonstrated that HMGA1 enhanced *Visfatin* and *VEGF* gene expression in human embryonic kidney (HEK) 293 cells in hypoxic conditions, whereas HMGA1 knockdown in differentiated 3T3-L1 adipocytes reduced these effects. Reporter gene analysis showed that *Visfatin* and *VEGF* transcriptional activity was increased by the addition of either HMGA1 or HIF-1 and even further by the combination of both factors. As demonstrated by chromatin immunoprecipitation in intact cells, HMGA1 directly interacted with the *VEGF* gene, and this interaction was enhanced in hypoxic conditions. Furthermore, as indicated by co-immunoprecipitation studies, HMGA1 and HIF-1 physically interacted with each other, supporting the notion that this association may corroborate a functional link between these factors. Therefore, our findings provide evidence for molecular cross-talk between HMGA1 and HIF-1, and this may be important for elucidating protein and gene networks relevant to obesity.

## Introduction

Obesity is a pathological condition often associated with insulin resistance, type 2 diabetes, cardiovascular disease, and even certain types of cancers (1, 2). Because of its alarming rise in incidence, and consequences in health care, interest in understanding the pathophysiological role of adipose tissue has consistently increased in the last decades. A major, relatively recent advancement in this field has been the concept that adipose tissue is not just an inert reserve of lipids, but a source of biomolecules, collectively called adipocytokines, with important implications in insulin sensitivity, inflammation, and angiogenesis (3–5). As adipocytokines are differentially expressed in the adipose tissue of obese versus lean individuals, a pro-inflammatory, insulin resistant, pro-atherogenic pattern typically prevails in the obese state. For example, it has been recognized that, in obesity, hypertrophic adipocytes produce monocyte chemotactic protein-1 (MCP-1), a factor favoring the infiltration of macrophages into fat tissue, while these immune cells produce pro-inflammatory cytokines, including the tumor necrosis factor α (TNFα), which sustain, in turn, adipose cell dysfunction (6, 7). However, the initial events leading to these changes in adipose tissue are still poorly understood.

An important issue is that the adipose tissue from obese individuals may become poorly oxygenated as adipocytes become larger, and distant from the vasculature (8–12). Hypoxia-inducible factors (HIFs) are important mediators of cellular adaptive response to hypoxia and play a role in the regulation of genes implicated in anaerobic metabolism, cell growth and survival, angiogenesis, and immune response (13, 14). In this regard, a master regulator that mediates hypoxic response is the HIF-1, a heterodimer constituted by α and β subunits. While HIF-1β is a constitutively expressed subunit, HIF-1α is continuously translated and degraded through ubiquitination under normoxic conditions (15, 16). In contrast, in hypoxic conditions, HIF-1α accumulates in the cytosol, being subsequently translocated into the nucleus, where it dimerizes with the HIF-1β subunit. As a transcription factor, the HIF-1α/β dimer binds to hypoxia responsive elements located within O_2_-regulated genes (17, 18), modulating a wide range of adaptive responses at low oxygen conditions, including glucose utilization, angiogenesis, apoptosis, extracellular matrix remodeling, and inflammation (12–14, 19, 20). Several other transcription factors have been recognized to be implicated in this scenario, including NF-kB, C/EBP homologous protein (CHOP), and the cAMP response element binding protein (CREB), all of which may interact and cooperate with HIF-1, in particular in the context of inflammation (21–23).

As reported (9), over thousand genes change their expression in adipose tissue hypoxia. However, how HIF-1 acts in the regulation of fat tissue-specific target genes, and which molecular partners are involved, need to be further characterized. We have hypothesized that the high-mobility group protein AT-hook 1 (HMGA1) may be a player in the gene expression networks that involve HIF-1 in adipose tissue. HMGA1 is an architectural transcription factor that functions as a dynamic regulator of chromatin structure (24–26), and is implicated in a variety of biological processes, including development, tumorigenesis, inflammation, and metabolism (25, 27–30). Several evidences have demonstrated a role for HMGA1 in the development of adipose tissue, both in pathological and physiological conditions. *HMGA* (*HMGA1* and *HMGA2*) gene rearrangements, due to chromosomal translocations, have been described in human benign neoplasias of mesenchimal origin, including lipomas (31), whereas a truncated form of HMGA1 has been reported to induce proliferation in 3T3-L1 preadipocytes (32). In a more physiological context, it has been reported that *in vitro*, in 3T3-L1 cells, HMGA1 is highly expressed during the first phases of adipose cell differentiation, while its deficit precludes terminal adipocyte conversion (27). Coherently, *in vivo*, in transgenic mice, HMGA1 overexpression in adipose tissue impairs adipogenesis and reduces fat mass by upregulating pre-adipocyte gene markers, and downregulating genes involved in adipocyte differentiation (33). At a molecular level, by binding to AT-rich sequences in the promoter region of the gene, HMGA1 modulates gene transcription either facilitating the binding of other transcription regulators to DNA, or interacting with other transcription factors that directly influence gene transcription (24, 34, 35). For example, HMGA1 has been shown to physically and/or functionally interact with NF-kB, PPARγ, and CEBP/β (36–38), which are abundantly expressed in adipose tissue. Previous findings indicate that hypoxia induces HMGA1 expression *in vitro*, and that this response is evolutionarily conserved (39, 40). However, up to now, a relationship between HMGA1 and HIF-1 has not been reported and the examination of this link could give further insights into our understanding of the regulation of genes involved in adipogenesis and obesity.

In the present study, we investigated two known HIF-1 target genes: the vascular endothelial growth factor (*VEGF*) gene and the *Visfatin* gene, both of which are normally expressed in adipose tissue (41–44). We show that HMGA1 physically interacts with HIF-1, and this interaction is required for proper transcription of these genes.

## Materials and Methods

### Cell Cultures

HEK-293 cells and 3T3-L1 mouse fibroblasts were cultured in Dulbecco’s modified Eagle medium (DMEM) (Sigma Aldrich) supplemented with 10% fetal bovine serum, 2 mM glutamine, penicillin (100 U/ml), and streptomycin (100 μg/ml), in a humidified 37°C, 5% CO_2_ incubator. Differentiation of 3T3-L1 fibroblasts into adipocytes was induced as described previously (45). Hypoxic conditions were obtained by placing cells for 24 h at 2% O_2_ in a hypoxic chamber (New Brunswick Galaxy 48R, Eppendorf), or by chemical induction with 100 μM cobalt chloride (CoCl_2_).

### Plasmids, Small Interfering RNA, and Transient Transfection

Reporter plasmids were as follows: p2025-Visfatin-Luc (a gift from A. Fukuhara, Osaka University, Osaka, Japan) and VEGF 2.6-Luc (a gift from N. Sheehy, University College Dublin, Dublin, Ireland). Expression plasmids were as follows: pcDNA3/HA-HMGA1 (32) and pcDNA3.1/HA-HIF-1α (a gift from G.L. Semenza, Johns Hopkins University, Baltimore, USA). For gene silencing experiments, a pool of three target-specific 20–25 nt siRNAs targeting mouse HMGA1 (Santa Cruz Biotech) was used. In all knockdown experiments, cells were transfected with 250 pmol of HMGA1 siRNA in six-well plates, and incubated without further treatment for 72 h before being used in subsequent analyses. Cell transient transfections were carried out using the Lipofectamine 2000 method (Invitrogen), and luciferase activity was assayed 48 h later, using the dual-luciferase reporter assay system (Promega).

### Real-Time PCR

For quantitative RT-PCR (qRT-PCR), total cellular RNA was extracted from 3T3-L1 cells using the RNAqueous-4PCR kit (Ambion), subjected to DNase treatment, and cDNAs were synthesized from 1 μg of total RNA using the RETROscript first strand synthesis kit (Ambion). Primers for mouse *HMGA1, Visfatin, VEGF*, and *Rps9* were designed according to sequences from the GenBank database. A real-time thermocycler (Eppendorf Mastercycler ep realplex ES) was used to perform qRT-PCR. SYBR Green fluorescence was measured, and relative quantification was made against the *Rps9* cDNA used as an internal standard. All PCR reactions were carried out in triplicates.

### Western Blot and Co-Immunoprecipitation Studies

Western blots were performed in nuclear extracts from 3T3-L1 and HEK-293 cells in both normoxic or hypoxic conditions. The antibodies used for these studies were: anti-HIF-1α (NovusBio), anti-Sp1, anti-HMGA1, and anti-Lamin A/C (SantaCruz) polyclonal antibodies. For co-immunoprecipitation studies, 200 μg of nuclear extracts from 3T3-L1 adipocytes or HEK-293 cells were mixed overnight with 5 μg of anti-HMGA1 antibody, as reported previously (38). Protein A Sepharose beads (GE Healthcare) were added for 90 min with rotation at 4°C. Antibody coupled protein A beads were washed twice with phosphate-buffered saline (PBS), proteins dissolved in Laemmli buffer and analyzed by SDS-PAGE and immunoblotting, using the polyclonal HIF-1α-specific antibody.

### Chromatin Immunoprecipitation

Chromatin Immunoprecipitation was performed in 3T3-L1 cells as described previously (35). As soon as the cells reached full differentiation, hypoxia was induced by treating cells with O_2_ 2%, 24 h. Then, cells were washed with PBS and fresh DMEM was added. DNA–protein complexes were cross-linked by adding formaldehyde for 10 min at room temperature, followed by blocking with glycine for 2 min. Cells were washed twice with cold PBS and lysed on ice using SDS lysis buffer (1% SDS, 10 mM EDTA, 50 mM Tris pH 8). Chromatin samples were sonicated on ice and the formaldehyde-fixed DNA–protein complexes were immunoprecipitated with anti-HMGA1 antibody, and sequence-specific primers for the mouse *Vegf* (and *Visfatin*) gene promoters were used for PCR amplification of immunoprecipitated DNA, using PCR ready-to-go beads (GE Healthcare). For the mouse *Vegf* gene, *Vegf*-specific primers (for 5′-GCT CTC TCT GAC CGG TCT CT-3′; rev 5′-GCA GAC TAT TCA GCG GAC TCA-3′) amplified a 270 bp region from −922 to −652 bp upstream of the ATG start site, which encompasses the consensus site for HMGA1. PCR products were electrophoretically resolved on 1.5% agarose gel and stained with ethidium bromide staining solution. To improve the reliability of comparisons within multiple experiments, we performed also qRT-PCR to amplify ChIP-ed DNA samples and to compare the amount of each with total input DNA used for each immunoprecipitation. Primers used were as above, while dissociation curves were analyzed to verify the quality of the amplicon and to exclude the presence of primer dimers.

### Statistical Analysis

All calculations were performed with SPSS 20.0 statistical software (SPSS Inc.). The non-parametric Mann–Whitney test was used for comparisons of data with a control, as foreseen in the Dunnett test approach (46). Results are shown as mean ± SE. A *p*-value <0.05 (two-tailed) was considered significant.

## Results

### Hypoxia and HMGA1 Increase *Visfatin* and *VEGF* Gene Expression

**Figure 1:**
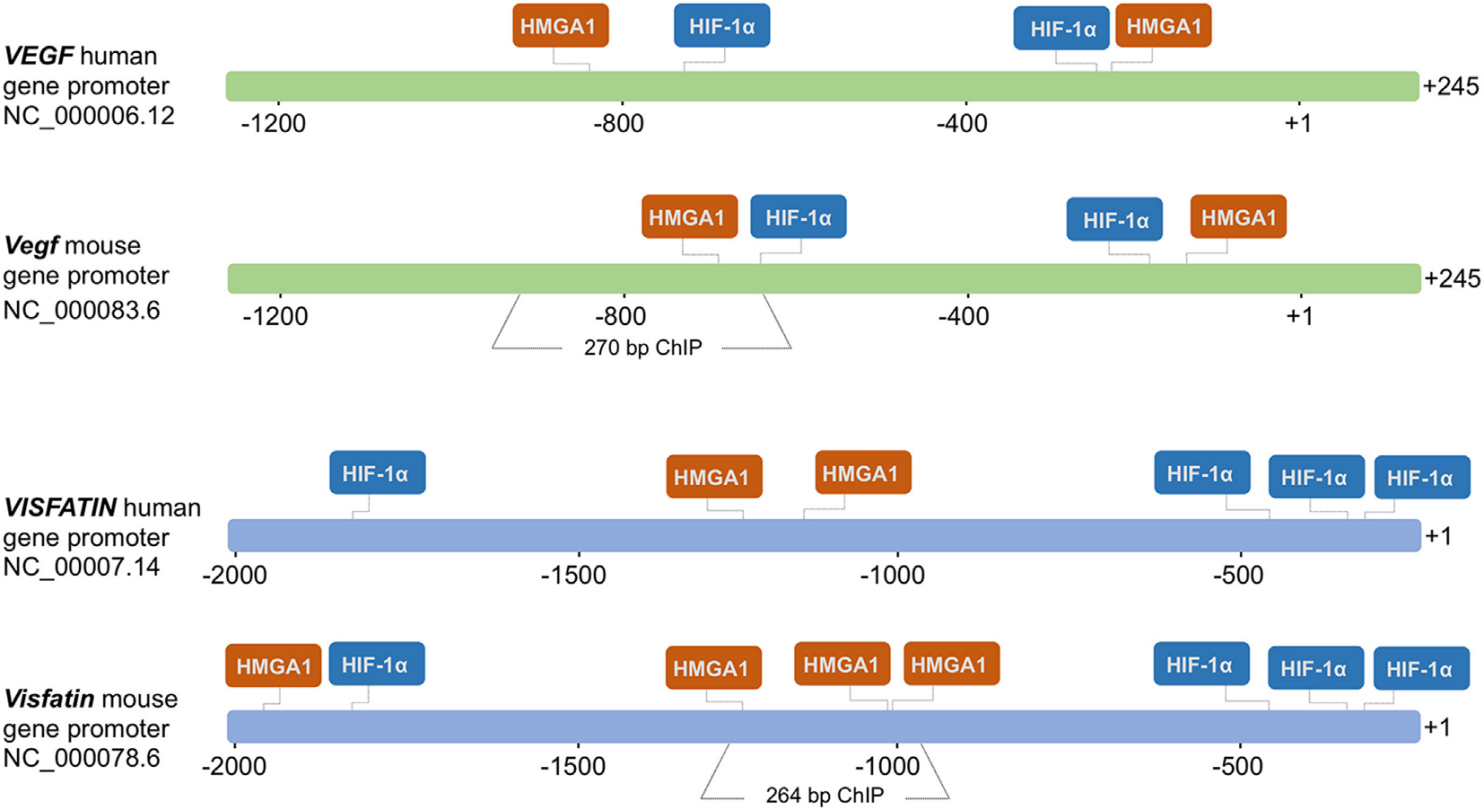
**Schematic representation of the human and mouse *VEGF* and *Visfatin* promoter regions**. HMGA1- and HIF-1α-binding sites are shown in each region. The numbers indicate positions in base pairs relative to the transcriptional start site (+1). Mouse *Vegf* and *Visfatin* promoter regions encompassing binding sites for HMGA1 and used in ChIP are indicated.

To test the hypothesis that HMGA1 could play a functional role in the transcriptional regulation of genes activated in hypoxic conditions, we first examined two genes, *VEGF* and *Visfatin*, which are both naturally expressed in adipose cells, and regulated by hypoxia. HMGA1 and HIF-1 consensus binding sites within the regulatory region of both these genes were identified by using the TRANSFAC database searched with MatInspector as informatic support (version 8.1, Genomatix, http://www.genomatix.de/). As shown in Figure 1, bioinformatic analysis predicted putative binding sites for HIF-1 and HMGA1 nuclear proteins in both human and mouse *VEGF* and *Visfatin* gene promoters.

**Figure 2:**
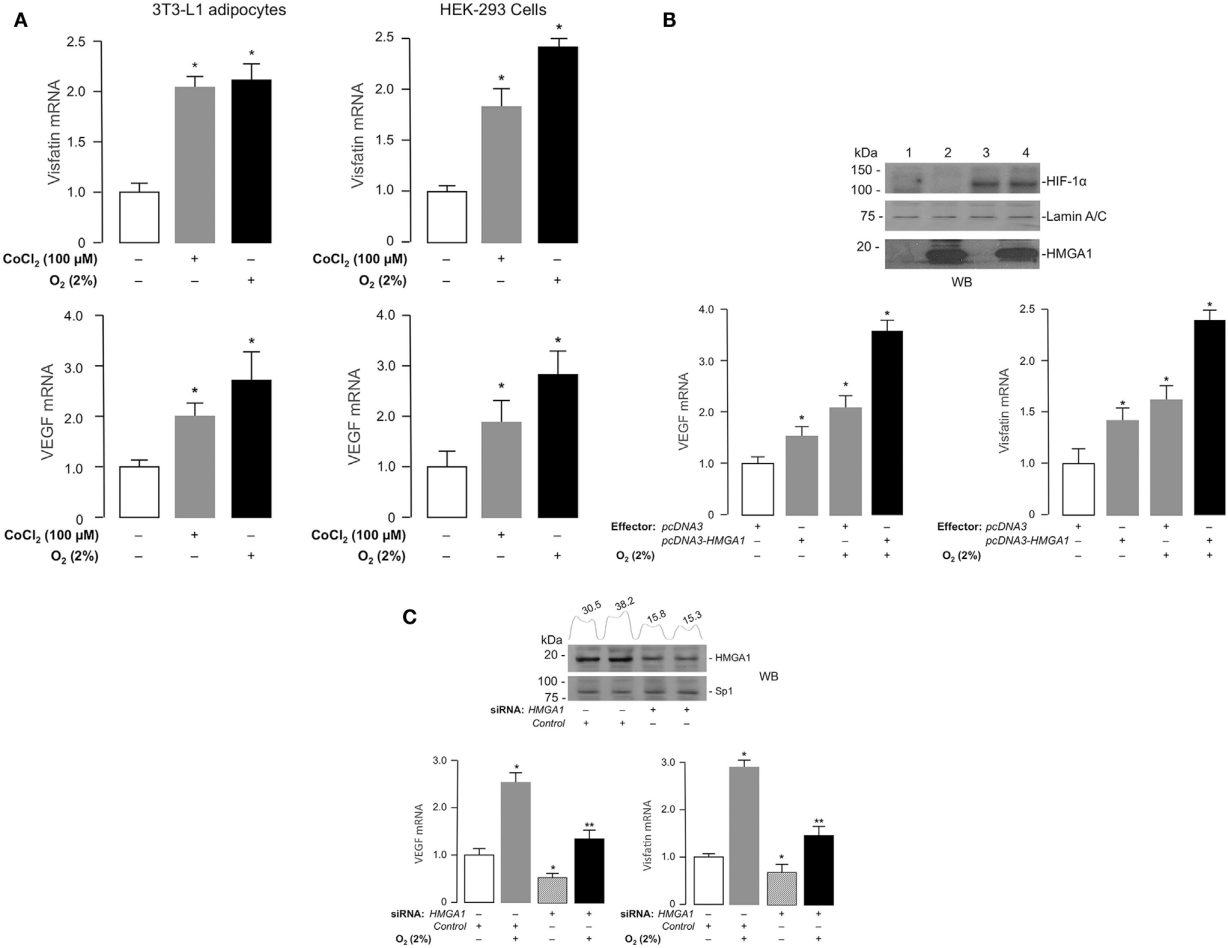
**Effects of hypoxia and HMGA1 on *Visfatin* and *VEGF* gene expression**. **(A)** 3T3-L1 adipocytes (left) and HEK-293 cells (right) were treated with CoCl_2_ or incubated under hypoxic conditions (O_2_ 2%) for 24 h. *Visfatin* and *VEGF* mRNA were measured by RT-PCR. Results are the means ± SE of triplicates from three independent experiments. Data are shown as fold of increment versus controls (white bars), which are assigned a value of 1. *p < 0.05 versus controls. **(B)** HEK-293 cells were transfected with HMGA1 plasmid (1 μg) under normoxic or hypoxic (O_2_ 2%) conditions. After 48 h, *VEGF* and *Visfatin* mRNA were measured by RT-PCR. Results are the means ± SE of triplicates from three independent experiments. Data are shown as fold of increment versus controls (white bars), which are assigned a value of 1. *p < 0.05 versus controls. Representative Western blots (WB) of HIF-1α and HMGA1 are shown for each experimental condition. Lanes: 1, control (pcDNA3 empty vector); 2, pcDNA3–HMGA1 effector vector; 3, pcDNA3 empty vector plus hypoxia; 4, pcDNA3–HMGA1 effector vector plus hypoxia. Lamin A/C, control of protein loading **(C)** 3T3-L1 adipocytes were incubated in the absence or presence of siRNA targeting HMGA1 (250 pmol) under normoxic or hypoxic (O_2_ 2%) conditions. *VEGF* and *Visfatin* mRNAs were measured by RT-PCR. Results are the means ± SE of triplicates from three independent experiments. *p < 0.05 versus controls (white bars); **p < 0.05 versus hypoxic conditions in the presence of scrambled siRNA (control, gray bars). Representative WBs of HMGA1 and Sp1 (this latter used as a control) from nuclear extracts of 3T3-L1 cells, either untreated or treated with siRNA targeting HMGA1, are shown. Densitometric slot blot analysis, using the ImageJ software program, is shown for HMGA1. Numbers on the peaks are the size of the corresponding slot as a percentage of the total size of the slots.

We next tested *Visfatin* and *VEGF* gene expression, and their response to hypoxia, in murine differentiated 3T3-L1 adipocytes, which express high levels of HMGA1 protein, and in human HEK-293 cells, a cell line ideally suited for studying the effects of HMGA1 on transcription since it does not express appreciable amounts of HMGA1. As shown by qRT-PCR, *Visfatin* and *VEGF* cDNAs were detectable in these cell lines in normal growth conditions, whereas both genes were further activated in cells under hypoxic conditions, as obtained by incubating cells in 2% O_2_ or when exposed to chemical hypoxia with CoCl_2_ (Figure 2A). When HMGA1 was overexpressed into HEK-293 cells, the expression of both *VEGF* and *Visfatin* genes increased in both normoxic and hypoxic conditions (Figure 2B). Conversely, knockdown of endogenous HMGA1 with HMGA1-specific siRNA inhibited *Vegf* and *Visfatin* gene expression in 3T3-L1 adipocytes in normoxic and hypoxic conditions (Figure 2C), thereby indicating a role for HMGA1 in the regulation of these hypoxia-inducible genes, and suggesting that HMGA1 is required for full activation of these genes during hypoxia.

### Transcriptional Regulation of *Visfatin* and *VEGF* Genes by HMGA1 and HIF-1

**Figure 3:**
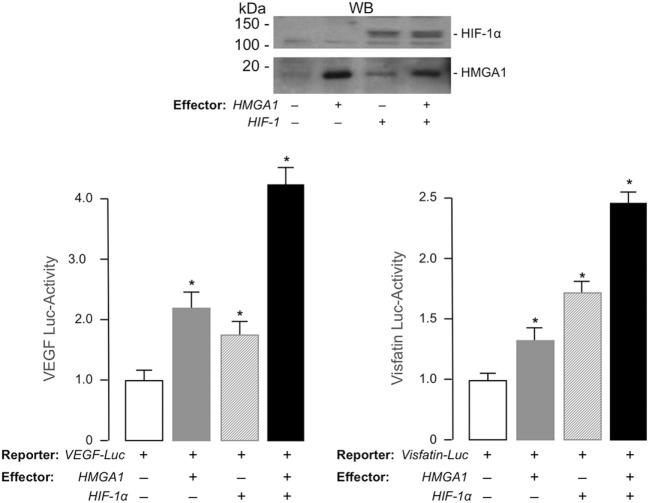
**Functional activity of HMGA1 and HIF-1α on *VEGF* and *Visfatin* gene transcription**. HEK-293 cells were cotransfected with 1 μg of human VEGF-Luc or 100 ng of mouse Visfatin-Luc reporter plasmid, in the absence or presence of effector vectors for HMGA1 and HIF-1α (1 μg each), either alone or in combination. Data represent the means ± SE for three separate experiments. Values are expressed relative to the VEGF- and Visfatin-Luc activities obtained in transfections with the reporter vector alone (control, white bars), which is assigned an arbitrary value of 1. *p < 0.05 versus control. Representative WBs of HMGA1 and HIF-1α from nuclear extracts of HEK-293 cells, either untransfected or transfected with human HMGA1 or HIF-1α expression vectors, are shown.

To explore the possibility that *Visfatin* and *VEGF* genes were transcriptionally regulated by a functional cooperation between HIF-1 and HMGA1, we transiently transfected HEK-293 cells with luciferase reporter plasmids containing the full-length sequence of the mouse *Visfatin* or the human *VEGF* gene promoter, in the absence or presence of recombinant plasmids expressing either HMGA1, HIF-1α, or both. As shown in Figure 3, forced expression of HMGA1 or HIF-1α in HEK-293 cells induced *Visfatin* and *VEGF* gene promoters, while concomitant overexpression of HMGA1 and HIF-1α yielded a synergistic increase in both *Visfatin* and *VEGF* promoter activities, thus indicating that, in hypoxic conditions, HMGA1 is required for full transactivation of these genes by HIF-1.

### HMGA1 and HIF-1 Protein–Protein Interaction and ChIP

**Figure 4:**
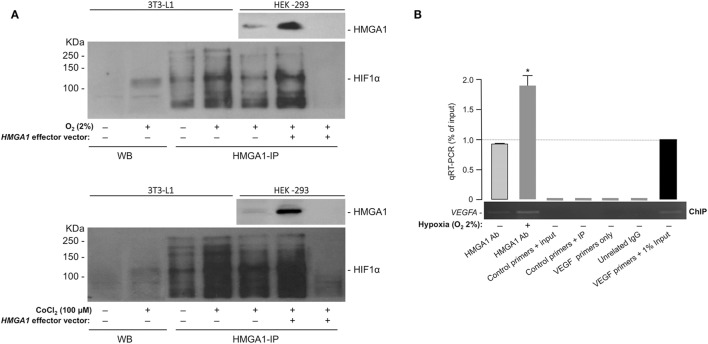
**HMGA1 and HIF-1 protein–protein interaction and ChIP**. **(A)** HIF-1α protein expression as shown by WB from 3T3-L1 adipocytes nuclear extracts in both normoxic or hypoxic (O_2_ 2%, upper panel; CoCl_2_ 100 μM, lower panel) conditions. Immunoprecipitation of HIF-1α from 3T3-L1 adipocytes and HEK-293 cell nuclear extracts under normoxic and hypoxic conditions, by using an anti-HMGA1 antibody (HMGA1-IP), followed by immunoblotting with an anti-HIF-1α specific antibody is shown in both panels. A negative control of immunoprecipitation (in the absence of HMGA1 antibody) is shown in the last right lane of each panel. Representative immunoblots are shown for each condition. Immunoprecipitations of HMGA1 followed by immunoblot analyses with the anti-HMGA1 antibody are shown in hypoxic HEK-293 cells, either untransfected or transfected with the HMGA1 effector vector. **(B)** ChIP analysis of the mouse *Vegf* promoter gene in 3T3-L1 adipocytes under normoxic and hypoxic (O_2_ 2%) conditions, using the anti-HMGA1-specific antibody (Ab). A representative ChIP assay is shown, together with qRT-PCR of ChIP-ed samples. Results are the mean ± SE for three independent experiments. *p* < 0.05 versus control (slashed bar).

The above results were further supported by protein–protein interaction experiments in whole 3T3-L1 adipocytes and HEK-293 cells, in both of which hypoxia was induced by either oxygen-deprivation in hypoxia-chamber or by the chemical hypoxia-mimicking agent CoCl_2_. While confirming that the HIF-1α protein product was indeed induced in nuclear extracts from 3T3-L1 cells in response to hypoxia (Figure 4A), we investigated whether HMGA1 and HIF-1 proteins physically interacted with each other in the context of the intact cell by performing co-immunoprecipitation studies. As shown in Figure 4A, immunoprecipitation of HMGA1 in nuclear extracts from 3T3-L1 adipocytes exposed to normoxia or hypoxia, followed by Western blot analysis for HIF-1α, revealed a protein-specific band, which migrated in a position corresponding to the size of HIF-1α, whose intensity considerably increased with hypoxia. HMGA1-HIF1 protein–protein interaction in hypoxia was substantiated in co-immunoprecipitation experiments using HMGA1 overexpressing HEK-293 cells, a cell line that normally produces barely amounts of HMGA1 (Figure 4A). Overall, these results indicate that HMGA1 and HIF-1 physically interact *in vivo* in intact cells, thereby suggesting that this step may represent an important prerequisite for the functional interplay between HMGA1 and HIF-1 in hypoxia.

These studies were complemented by ChIP in differentiated 3T3-L1 adipocytes. We found that HMGA1 bound to the endogenous *VEGFA* chromosomal locus, and that this binding was enhanced after incubation of cultured cells in hypoxic conditions (2% O_2_) (Figure 4B). Of note, no binding of HMGA1 to the *Visfatin* gene was observed in parallel experiments with 3T3-L1 cells (not shown), suggesting that HMGA1 may influence *Visfatin* gene transcription through alternative mechanisms, not necessarily involving direct binding of HMGA1 to DNA. A similar behavior has been reported for other genes, such as the *Leptin* and the interferon beta (*IFN-*β) genes (27, 47).

## Discussion

Cellular oxygen sensing is known to be mostly mediated by specific nuclear transcription factors, among which HIF-1 occupies a pivotal position. How hypoxia, through HIF-1, impacts, at the molecular level, fat tissue-specific target genes and gene networks, is only partly understood. It is plausible that in most cases, HIF-1 modulates the transcriptional activity of target genes by regulating and/or interacting with other known ubiquitous or tissue-specific nuclear factors. In this regard, in adipose tissue, HIF-1 prevents adipogenesis by inhibiting PPARγ2 and C/EBPβ gene expression (48). In addition, HIF-1 is regulated by and interacts with NF-kB, a transcription factor that plays a pivotal role in the regulation of the inflammatory and immune responses (49).

High-mobility group AT-hook 1 shares with HIF-1 similar effects on adipogenesis and inflammation by inhibiting adipose cell terminal differentiation and by promoting the expression of proinflammatory cytokines. For example, an involvement of both HMGA1 and HIF-1 has been reported for the regulation of the *Leptin* and *Pref-1* genes in the early phase of adipogenesis (9, 27, 33). On the other hand, cooperation among molecular partners common to HMGA1 and HIF-1, such as NF-kB, has been reported for the upregulation of genes involved in the immune response/inflammation, including *IL-6* (9, 50), *IFN-*β (36, 51), and several cytokine and adhesion molecule genes (9, 52). Furthermore, in the context of the cyclooxygenase-2 (*COX-2*) gene promoter, hypoxia has been shown to induce HMGA1 as part of a hypoxia-induced enhanceosome that encompasses, among others, the NF-kB transcription factor, and helps to promote transcription of *COX-2* (39). Despite of these findings, however, no evidence of a link between HIF-1 and HMGA1 has been previously searched and identified.

Herein, we were interested to investigate whether HIF-1 cooperates with HMGA1 in the functional regulation of adipose tissue-specific gene expression. Using *VEGF* and *Visfatin* as established HIF-1-target genes in 3T3-L1 adipocytes, we demonstrate that HMGA1 functionally interacts with HIF-1, thereby inducing transcriptional activation of both target genes. Our findings indicate that hypoxia-induced transcription of *VEGF* and *Visfatin* requires HMGA1 for maximal transactivation of these genes by HIF-1, and that repression of HMGA1 expression adversely affects HIF-1 function. Using ChIP, we demonstrated that HMGA1 can directly interact with the endogenous *VEGF* promoter. Intriguingly, this was not the case for the *Visfatin* gene, for which no HMGA1–DNA binding was observed in ChIP assays, although multiple putative binding sites for HMGA1 were detected within the *Visfatin* promoter. One possibility for the functional interrelationship between HIF-1 and HMGA1 in the context of the *Visfatin* gene, during hypoxia, is that HMGA1 may act through protein–protein interaction mechanism, without direct binding to DNA. This is the case, for example, of the *Leptin* gene (27) and *IFN-*β gene (47), for which HMGA1 strongly potentiates transactivation by C/EBPβ and NF-kB, respectively, through direct protein–protein contacts independent of DNA. Alternatively, HMGA1 could recruit component(s) of the basal transcription machinery toward protein–DNA complexes, thereby promoting transcription, with little, if any, specificity for the putative target DNA sequence (53). In support of these non-canonical mechanisms, physical association between HIF-1 and HMGA1 has been observed in our study, and this association may be a prerequisite for the HIF-1 effects in hypoxia.

While in adipose tissue the cooperation between HIF-1 and HMGA1 may coherently recapitulate their parallel roles in adipogenesis and inflammation, other important biological effects, such as those on insulin action and glucose metabolism, in which HIF-1 and HMGA1 act in an apparently divergent manner (i.e., insulin resistance versus insulin sensitivity), deserve future searches and explanations. Studies in adipose tissue and cells have shown a role of hypoxia in insulin resistance (9, 20) and in the impairment of insulin action (54), while adipose tissue-specific disruption of HIF-1 in mice fed with high-fat diet improves insulin sensitivity (55). In contrast, HMGA1 plays a positive role in insulin biosynthesis and action (29, 35, 56), as well as in the transcription of a number of glucose metabolism-related genes (29, 30, 38, 57). Thus, whereas *HMGA1* gene defects associate with insulin resistance and diabetes in humans and mice (29, 58, 59), HMGA1 overexpression in adipose tissue prevents insulin resistance in mice (33). Therefore, studies aimed at investigating cross-talks between HMGA1, HIF-1, and their molecular partners at the level of gene transcription may help explaining the divergent role of these factors in insulin response.

Overall, we believe our findings may contribute to elucidate the molecular mechanisms underlying hypoxia-induced gene regulation and expression in adipose cells. The identification of gene expression networks involved in adipose cell dysfunction represents a challenging field of research that may improve our knowledge on the pathophysiology of obesity and obesity-related disorders.

## Author Contributions

SM, AEL, and BA performed the experiments; DF conceived the work and wrote the first draft of the manuscript; EC and RH supervised PCR and immunoblot experiments and critically read the manuscript; AB critically revised and edited the manuscript. All the authors have approved the submitted version.

## Conflict of Interest Statement

The authors declare that the research was conducted in the absence of any commercial or financial relationships that could be construed as a potential conflict of interest. The reviewer (ZM) and handling Editor declared their shared affiliation, and the handling Editor states that the process nevertheless met the standards of a fair and objective review.
